# Cilium Expression Score Predicts Glioma Survival

**DOI:** 10.3389/fgene.2021.758391

**Published:** 2021-11-19

**Authors:** Srinivas Rajagopalan, Amartya Singh, Hossein Khiabanian

**Affiliations:** ^1^ Center for Systems and Computational Biology, Rutgers Cancer Institute of New Jersey, Rutgers University, New Brunswick, NJ, United States; ^2^ Department of Pathology and Laboratory Medicine, Rutgers Robert Wood Johnson Medical School, Rutgers University, New Brunswick, NJ, United States

**Keywords:** cilium, glioma, glioblastoma, risk score, survival, gene expression, biclustering

## Abstract

The accurate classification, prognostication, and treatment of gliomas has been hindered by an existing cellular, genomic, and transcriptomic heterogeneity within individual tumors and their microenvironments. Traditional clustering is limited in its ability to distinguish heterogeneity in gliomas because the clusters are required to be exclusive and exhaustive. In contrast, biclustering can identify groups of co-regulated genes with respect to a subset of samples and vice versa. In this study, we analyzed 1,798 normal and tumor brain samples using an unsupervised biclustering approach. We identified co-regulated gene expression profiles that were linked to proximally located brain regions and detected upregulated genes in subsets of gliomas, associated with their histologic grade and clinical outcome. In particular, we present a cilium-associated signature that when upregulated in tumors is predictive of poor survival. We also introduce a risk score based on expression of 12 cilium-associated genes which is reproducibly informative of survival independent of other prognostic biomarkers. These results highlight the role of cilia in development and progression of gliomas and suggest potential therapeutic vulnerabilities for these highly aggressive tumors.

## Introduction

Brain cancers are broadly categorized into low- or high-grade tumors. Low-grade gliomas (LGG) consist of grades II and III and are categorized as astrocytoma or oligodendroglioma because they can initiate from astrocytes or oligodendrocytes, respectively (Louis et al., 2016). Grade IV tumors are categorized as glioblastoma (GBM) and are amongst the most common and aggressive malignant brain cancers, developing either as a primary tumor without a known precursor, or as a secondary tumor through progression from a low-grade glioma (Davis, 2016). The clinical outcome for all gliomas is grim. The mean duration of survival after diagnosis for low-grade glioma patients is around 7 years (Poon et al., 2020); nevertheless, almost all low-grade gliomas eventually progress to a higher-grade tumor, which have an average survival time of less than 2 years (Poon et al., 2020).

In the past, brain tumors were assessed and classified based on histology. In 2016, the World Health Organization integrated molecular biomarkers into its classification process, enabling clinicians to implement an algorithmic approach towards glioma diagnosis and prognosis, resulting in greater objectivity, consistency, and reliability (Louis et al., 2016). For instance, low-grade gliomas have been classified according to the 1p and 19q loci co-deletion as well as the mutations in the *IDH1* and *IDH2* genes; tumors without these alterations have been shown to be clinically and molecularly similar to high-grade tumors (Claus et al., 2015). High-grade glioblastomas have been classified according to gene expression into three subtypes: classical (associated with high level *EGFR* amplification), proneural (associated with exclusive *PDGFRA* amplification), and mesenchymal (associated with high expression of genes linked to immune response and the tumor necrosis) (Olar and Aldape, 2014).

In addition to molecular characteristics of a tumor that determine its clonal evolution (Ceccarelli et al., 2016), tumor progression can also be modulated by cellular heterogeneity present within its microenvironment. In fact, gene expression patterns associated with non-tumor cells have been shown to have prognostic value for gliomas (Coussens and Werb, 2002). For example, the expression of a combination of immune genes were used to develop a prognostic, localized risk score. Tumors with high immune risk scores were mostly of the mesenchymal subtype, suggesting therapeutic benefit from immunotherapy for them (Cheng et al., 2016). Similarly, an expression signature of 20 genes involved in extracellular matrix organization was shown to be effective in predicting glioma survival and prognosis (Celiku et al., 2017). It has also been demonstrated that glioma cells foster synaptic connections with neurons when they invade normal neural circuitry (Venkatesh et al., 2019), providing a survival benefit to the brain tumor. However, these properties are not uniform amongst all gliomas; only certain types of glioma cells are able to interact with specific types of brain cells (Gao et al., 2020), suggesting the need for a finer characterization of brain tumors based on their interaction with the microenvironment.

In this study, we sought to characterize the heterogeneity in transcriptomic profiles of both normal and tumor brain tissues and to identify co-regulated gene expression signatures associated with prognosis in gliomas. To this end, we analyzed batch-corrected gene expression data from normal brain tissues from The Genotype-Tissue Expression (GTEx) (Consortium, 2013) study and primary gliomas from The Cancer Genome Atlas (TCGA) GBM and LGG cohorts (Ceccarelli et al., 2016). We used a biclustering algorithm called TuBA, which detected co-regulated genes within subsets of samples (Singh et al., 2019), and identified gene expression signatures associated with proximally located brain regions, as well as co-regulated genes aberrantly regulated in tumor samples. Specifically, we identified a signature related to cilium motility associated with particular regions in the brain that, when highly expressed in gliomas, was indicative of poor prognosis. Our single-cell analysis of patients with gliomas associated the cilium expression signature with the tumor cells. Finally, we devised a risk score predictive of survival and evaluated it in independent glioma cohorts after correcting for other clinically relevant biomarkers.

## Methods

Patient Cohorts

The Genotype-Tissue Expression (GTEx) RNA-seq data for normal brain and The Cancer Genome Atlas (TCGA) RNA-seq data for GBM and LGG cohorts were analyzed. Combined and batch-corrected normal brain tissue as well as brain tumor gene expression data [RSEM (Li and Dewey, 2011)] were obtained from the UCSC Xena Portal (http://xena.ucsc.edu; accessed April 2, 2021), including 1,136 GTEx samples, 509 TCGA LGG samples, and 153 TCGA GBM samples. Normal brain GTEx samples were annotated for their specific regions in the brain. We additionally obtained the log Transcripts Per Million (TPM) values for differential pathway analysis (described below).

To compare tumor purity between TCGA samples, ESTIMATE scores were obtained from Yoshihara et al. (2013), and immunohistochemistry (IHC) and LUMP (leukocytes unmethylation for purity) results were obtained from Aran et al. (2015). The Mann-Whitney *U* test was used to determine significant differences in purity between samples.

Normalized gene expression microarray data from the REMBRANDT study were also obtained from the Gene Expression Omnibus (GEO) Portal (accession ID: GSE108474) for 261 GBM and 269 LGG samples (Gusev et al., 2018). Phenotype and survival information was obtained for these patients through the Gliovis Portal (Bowman et al., 2017).

Unique molecular identifier (UMI) counts for 6,148 single-cell transcriptomes collected from 73 regions in 13 patients with glioma (3 grade II, 1 grade III, 8 grade IV, and 1 gliosarcoma) (Yu et al., 2020) were obtained from the GEO Portal (accession ID: GSE117891).

### Biclustering Analysis

The Tunable Biclustering Algorithm (TuBA) analyzes gene expression data to identify sets of co-expressed genes in subsets of samples (Singh et al., 2019). TuBA uses a proximity measure that connects gene pairs that have a significant number of samples shared between their top percentile sample sets (for high expression analysis) or bottom percentile sample sets (for low expression analysis). In this work, TuBA’s proximity measure was redefined using the Jaccard index to determine significance of samples overlapping in the genes’ percentile sets, which specifically resulted in faster computation time. TuBA’s biclustering parameters were chosen such that the total number of edges in graph was less than 500,000. We additionally added constraints for the minimum number of samples and genes in the biclusters. The following parameters were chosen for individual analyses presented in Results:

1. GTEx + TCGA GBM and LGG high expression analysis: top percentile cutoff of 3% with Jaccard Index of 0.33 (minimum number of genes = 31, minimum number of samples = 100)
2. GTEx + TCGA GBM and LGG low expression analysis: bottom percentile cutoff of 5% with Jaccard Index of 0.4 (minimum number of genes = 32, minimum number of samples = 100)
3. TCGA-only, GBM and LGG high expression analysis: top percentile cutoff of 5% with Jaccard Index of 0.4 (minimum number of genes = 30, minimum number of samples = 38)
4. TCGA-only, LGG high expression analysis: top percentile cutoff of 5% with Jaccard Index of 0.45 (minimum number of genes = 32, minimum number of samples = 30)
5. REMBRANDT high expression analysis: top percentile cutoff of 5% with Jaccard Index of 0.4 (minimum number of genes = 32, minimum number of samples = 30)

### Single-Cell Biclustering Analysis

In order to apply TuBA to single-cell RNA sequencing data, first, genes that had UMI count of zero in more than 97.5% of the cells were removed (Minimum population with non-zero counts were determined based on TuBA’s percentile set size parameter of 2.5% in this analysis). Following recommendation by Lause et al. (2021), the UMI counts were transformed to Pearson residuals defined by,

$$Z_{cg}=\left(X_{cg}-\mu_{cg}\right)/\surd \left(\mu_{cg}+\frac{\mu_{cg}^{2}}{\theta }\right)$$

where,

$$\mu_{cg}=\left(\frac{\sum_{i}X_{ig}}{\sum_{ij}X_{ij}}\right)\underset{j}{\sum }X_{cj}.$$

In the equations above, *c* refers to cell while *g* refers to gene and θ = 100. Gene-pairs with Jaccard index values greater than 0.2 were used to generate graphs that were then iteratively examined by TuBA to discover biclusters. The UMAP plots were generated using the Pearson residuals matrix corresponding to these 2,152 shortlisted genes, which had variances of their Pearson residuals > +1 standard deviation away from the mean of the variances of all the genes.

### Enrichment Analysis

Enrichr (version 3.0) was used for gene-set enrichment analysis (Xie et al., 2021). The Gene Ontology (GO) biological process subontology (GO-BP) (Gene, 2021) was used to evaluate functional relationships between the genes in each bicluster. Hypergeometric test after correction for false discovery rate (FDR) by the Benjamini-Hochberg method was used to evaluate the enrichment of clinical features for samples in each bicluster.

### Differential Gene and Pathway Analysis

Supervised differential pathway and gene-set expression between samples was performed using Gene-Set Variation Analysis (GSVA) (Hanzelmann et al., 2013), across the entire Reactome Pathway database (Jassal et al., 2020) based on log of TPM values. The Mann-Whitney *U* test with FDR correction was used to determine significant differences between GSVA’s pathway-and-sample-specific scores. We also used GSVA to calculate enrichment scores for gene-sets identified in TuBA’s biclusters in bulk and single-cell analyses. Differential gene expression analysis was performed using DESeq2 (Love et al., 2014).

### Cilium Risk Score

Cox models are used to assess the importance of predictor variables in a regression model for survival (Cox, 1972; Therneau and Grambsch, 2000). In order to develop risk scores based on expression of cilium genes and to correct for previously uncovered clinically covariates, a Cox regression model based on overall survival was developed (Simon et al., 2011). Using glmnet specifications in R, an alpha value–representing the elastic net mixing parameter–of one was used corresponding to pure LASSO regression (Friedman et al., 2010). The model was trained on the following factors: the co-regulated cilium genes identified by TuBA in the GTEx + TCGA analysis, *IDH1/2* mutation and 1p/19q co-deletion status, tumor grade, and patient age at diagnosis. In order to linearly combine the expression values of cilium genes into a risk score, a univariate model for each significant cilium gene was created and the coefficients from these models was used as coefficients for the linear combination.

All analyses were performed in R (version 4.1.0), including the ComplexHeatmap (Gu et al., 2016), survminer, and ggplot2 (Wickham, 2016) packages. All scripts are available at https://github.com/KhiabanianLab.

## Results

### Integrated Analysis of TCGA and GTEx Samples Reveals Distinct Gene Expression Signatures in Tumor and Normal Brain Tissue

We applied TuBA to batch-corrected RNA-seq data from 1,798 samples (1,136 normal brain from GTEx, 509 LGG and 153 GBM specimens from TCGA) and generated 102 biclusters for high-expressing (top 3% percentile) co-regulated genes (Methods). We assessed the associations with clinical and molecular information available for samples in the biclusters and performed pathway enrichment analysis for their sets of genes. In this combined GTEx + TCGA analysis, TuBA uncovered co-expression signatures that distinguished between glioma and normal brain tissues, where 57 biclusters were enriched in GTEx samples while 42 were enriched in TCGA samples (Fisher’s exact FDR <0.01).

**FIGURE 1:**
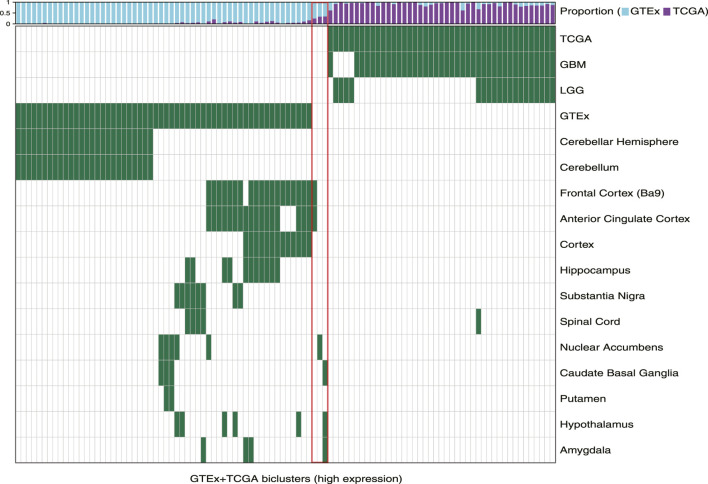
Biclusters from TuBA’s high expression analysis, either enriched in the GTEx normal tissue collected from different brain regions or enriched in the TCGA tumors with LGG or GBM subtypes. The bars show the proportion of GTEx vs. TCGA samples in each bicluster. The red box highlights the three biclusters with enrichment in neither TCGA nor GTEx samples, including the cilium bicluster (most left) with enrichment in amygdala, hypothalamus, and caudate normal tissue.

First, we observed that TuBA detected co-regulated genes that were expressed at higher levels in distinct regions of the brain relative to others. Of 57 GTEx-specific biclusters, 27 primarily contained samples collected from cerebellum and cerebellar hemisphere (Figure 1). The genes in these biclusters were associated with the GO-BP terms such as regulation of gene expression and regulation of cellular macromolecule biosynthetic process, and included those coding for zinc-finger proteins (Supplementary Table S1). The other 30 biclusters contained samples collected from multiple but distinct regions in the brain located proximally to each other. For example, we identified six biclusters enriched in caudate basal ganglia (Fisher’s exact FDR <0.01); three of these biclusters were also enriched in samples from the putamen, and four were enriched in samples from the nuclear accumbens. Caudate, putamen, and nuclear accumbens are all components of the basal ganglia, and therefore, are located next to each other. We observed a similar pattern for biclusters enriched in anterior cingulate cortex, frontal cortex, and cortex regions, as well as biclusters enriched in substantia nigra and spinal cord (Figure 1). The genes in GTEx-specific biclusters were associated with chemical synaptic transmission, ion transmembrane, and other neuropathological pathways (Supplementary Table S1). Of note, the genes in biclusters enriched in spinal cord tissue were also associated with immune-related GO-BP terms, such as neutrophil degranulation and neutrophil activation, highlighting transcriptional heterogeneity across the normal brain, which may be impacted by localized cellular environment.

Next, we observed that TCGA-specific biclusters contained samples with distinct tumor subtypes: 22 were enriched in GBM samples, four were enriched in LGG samples, and 16 contained both GBM and LGG sample. Pathway analysis of genes in the GBM-specific biclusters revealed significant associations with Wnt signaling, planar cell polarity, neutrophil mediated immunity, and protein N-linked glycosylation. Genes in the LGG-specific biclusters were associated with GO-BP terms such as positive regulation of telomere maintenance, axonogenesis, RNA splicing, and translation (Supplementary Table S1). Seven of 42 tumor-specific biclusters contained genes associated with DNA metabolic processes and mitotic cell cycle phase transition previously implicated in gliomas (Friedman et al., 2010). Four of these seven biclusters were exclusively enriched in GBM samples.

### Cilium-Associated Genes Are Expressed Highly in Both Tumor and Normal Brain Tissue

**FIGURE 2:**
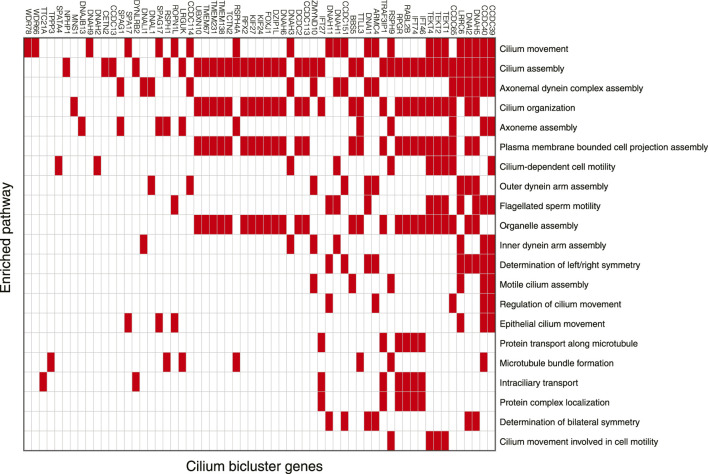
Genes in the cilium bicluster associated with top 20 significantly enriched GO-BP pathways. Full list of genes and enrichments are available in Supplementary Table S1.

Among 102 biclusters identified by TuBA, only three were neither enriched in the GTEx nor TCGA samples (Figure 1; red box). The GO-BP terms enriched for the genes in two of these biclusters were chemical synaptic transmission and postsynaptic and respiratory electron transport chain. The top-five GO-BP terms for 347 genes in the third bicluster, consisting of 73 GTEx and 40 TCGA samples, were cilium movement, cilium assembly, axonemal dynein complex assembly, and cilium organization. (Figure 2, Supplementary Table S1). Henceforth, we will refer to this bicluster as the “cilium bicluster.”

While the GTEx samples in the cilium bicluster were enriched in specific regions of the brain from the amygdala, hypothalamus, and caudate region (Figure 1), the TCGA samples were not enriched in any particular tumor subtypes: 12 were GBM, 28 were LGG; and while seven were *IDH1/2*-mutated and/or 1p/19q co-deleted, 32 were wild-type and one did not have subtype information.

### Downregulated Pathways Reflect Tumor and Normal Gene Expression Signatures

**FIGURE 3:**
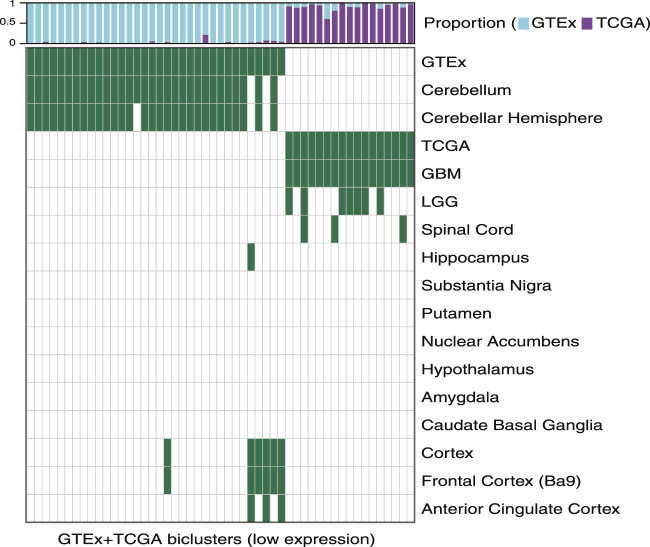
Biclusters from TuBA’s low expression analysis, either enriched in the GTEx normal tissue collected from different brain region or enriched in the TCGA tumors with LGG or GBM subtypes. The bars show the proportion of GTEx vs. TCGA samples in each bicluster. GTEx-specific biclusters were enriched in cerebellum, cerebellar hemisphere, cortex, frontal cortex, or anterior cingulate cortex, while all TCGA-specific biclusters were enriched in GBM tumors.

We used TuBA to generate biclusters consisting of co-regulated genes that had expressions in the bottom 5% of normal and tumor samples (Methods). Of the 51 biclusters obtained, 34 were enriched in GTEx normal samples and 17 were enriched in TCGA glioma samples (FDR <0.01). Corroborating the results from the high-expression analysis, the majority of the GTEx-specific biclusters (29 of 34) were enriched in samples from cerebellum or cerebellar hemisphere. Other five GTEx-specific biclusters were enriched in frontal cortex and cortex samples (Figure 3, Supplementary Table S2).

While seven of the TCGA-specific, low expression biclusters contained both LGG and GBM samples, all 17 were enriched in GBM tumors. The GO-BP terms associated with the genes in these biclusters suggested downregulation of axonogenesis, protein phosphorylation, anterograde *trans*-synaptic signaling, and histone modification in these tumors. In particular, one TCGA-specific bicluster contained genes that suggested downregulation of synaptic transmission, reflecting TuBA’s results for co-regulated higher expression of the genes associated with this pathway in normal brain tissue (Supplementary Table S2). Of note, samples in this bicluster significantly overlapped with those in 39 of 42 TCGA-specific high-expression biclusters (including 68% of tumors in the cilium bicluster), which highlighted common aberrantly regulated mechanism in gliomas despite their heterogeneity in upregulated pathways (Garofano et al., 2021).

### Single-Cell Analysis Suggests Cilium Expression Signature Arises From Tumor Cells

In order to investigate the cellular origin of cilium expression, we analyzed 6,148 single-cell transcriptomes collected from 13 patients with glioma, profiling 73 regions within the core of the tumors as well as those isolated from peritumoral sites (Yu et al., 2020). Yu *et al.* reported one patient (labeled GS13) with an *IDH*-wild-type GBM characterized by high expression of genes associated with motile cilium activities. In particular, they found that the cells exhibiting upregulation of these cilium motility associated genes belonged to the core tumor sites.

**FIGURE 4:**
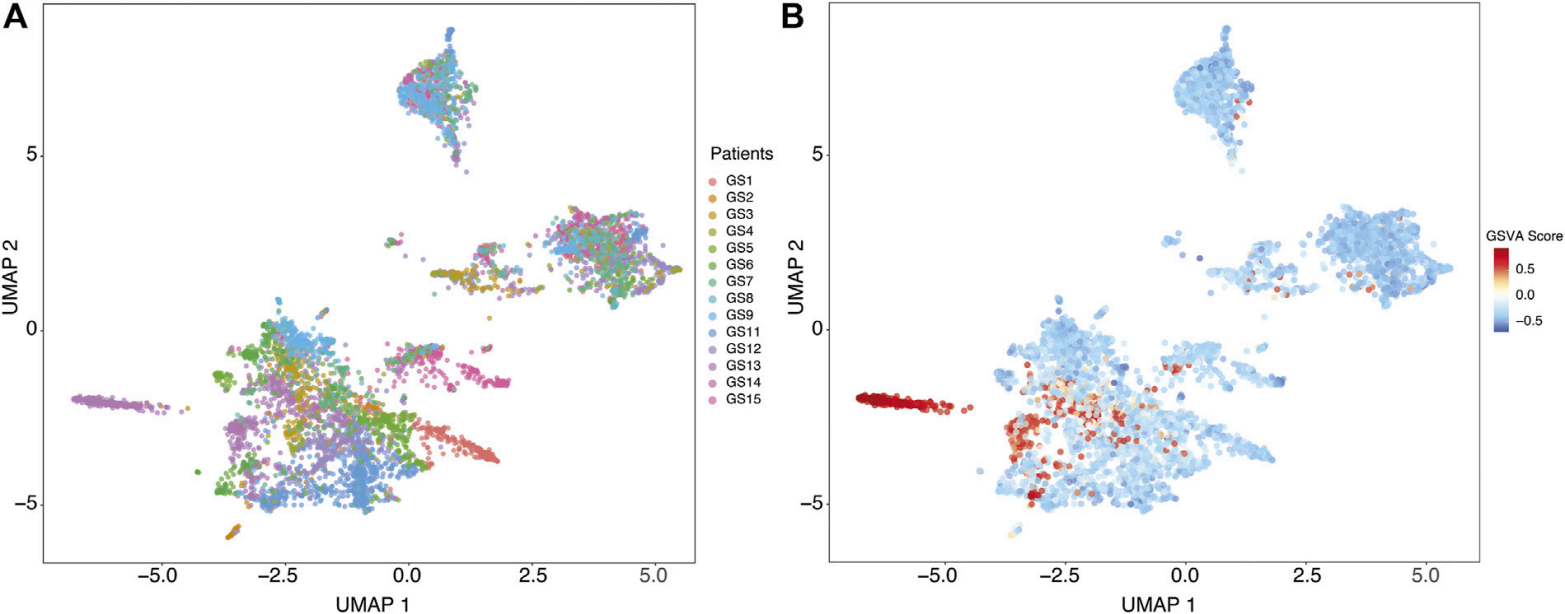
**(A)** Uniform Manifold Approximation and Projection (UMAP) of Pearson residuals across 13 patients. **(B)** GSVA enrichment scores in all cells across all patients identifies cells with upregulation (positive GSVA scores) of the cilium genes, dominantly in cells from GS13.

**FIGURE 5:**
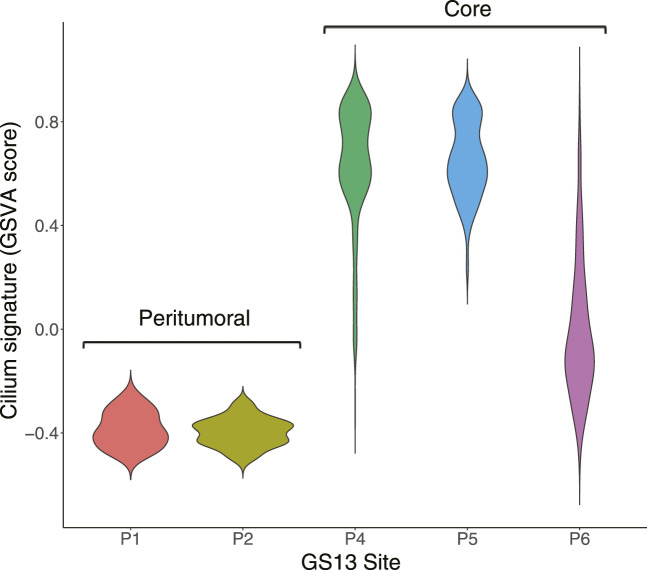
Distribution of GSVA enrichment scores for the cilium gene-set identified by TuBA shows a significantly higher expression in cells obtained from GS13’s core tumor sites P4, P5, and P6.

We identified distinct cell groups across the patients by reducing dimensionality using Uniform Manifold Approximation and Projection (UMAP) of Pearson residuals (Methods) (Figure 4A) consistent with the original findings. We then applied TuBA to single-cell expression profiles and obtained 51 biclusters, including Bicluster 1 comprising genes associated with cilium motility (Supplementary Table S3). Out of the 370 cells in this bicluster, 312 cells were from GS13. Moreover, all 312 cells from GS13 were collected from the core tumor sites –169 cells from site P4, 132 from P5, and 11 from P6. To evaluate and summarize the expression of the gene-set in Bicluster 1, we calculated the GSVA enrichment scores in all cells across all patients which showed upregulation (positive GSVA scores) of the cilium genes dominantly in GS13 (Figure 4B). In particular, cilium gene expression was significantly higher in cells obtained from GS13’s core tumor sites (P4, P5, P6), thereby highly suggesting tumoral origin of the cilium motility signature (Figure 5).

### High Cilium Expression Is Predictive of Poor Prognosis in Gliomas

To investigate the clinical relationship between co-regulated genes and tumors’ clinical features, we applied TuBA to only the TCGA GBM and LGG samples and obtained 67 biclusters, 40 of which were enriched in GBM and 13 were enriched in LGG samples. The TCGA-only biclusters were highly concordant with the GTEx + TCGA biclusters –99% of the former significantly overlapped with the genes and samples in the latter (Fisher’s exact FDR <0.05). Specifically, we again identified a bicluster with genes associated with cilium containing 57 LGG of varying histology and 40 GBM tumors. Of note, these samples included all 40 tumors found in the GTEx + TCGA cilium bicluster.

**FIGURE 6:**
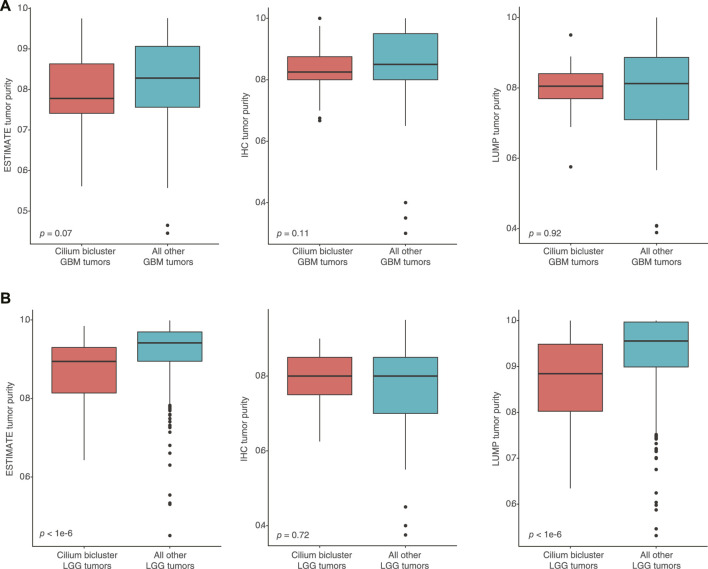
**(A)** No significant differences in computational and IHC-based purity assessments for GBM samples with vs. without cilium-associated expression. **(B)** Significant difference in computational purity estimates by ESTIMATE and LUMP for LGG tumors in the cilium bicluster compared to other LGG tumors, contrasting with no significance difference in their IHC-based purity estimates.

To assess whether high-cilium tumors had differential specimen tumor purity, we used estimates from IHC as well as computational analyses by ESTIMATE (based on expression of selected immune and stromal genes (Yoshihara et al., 2013)) and LUMP (based on methylated status of immune-specific CpG sites (Aran et al., 2015)). We did not find significant differences in purity assessments for GBM samples with vs. without cilium-associated expression. However, computational estimates for LGG tumors contrasted with IHC and suggested lower purity for LGG samples in the cilium bicluster (Figure 6).

**FIGURE 7:**
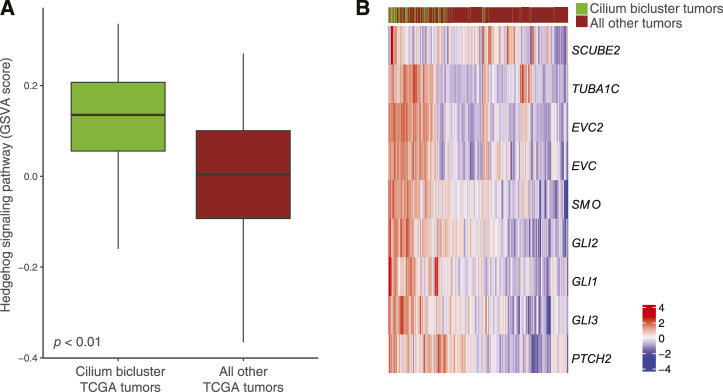
**(A)** Upregulation of the Hedgehog signaling pathway in TCGA tumors with cilium-associated expression based on the GSVA scores. **(B)** Differential expression of Hedgehog signaling genes in the cilium bicluster tumors vs. all other tumors.

Differential pathway analysis of tumors in the cilium bicluster against all other tumors using GSVA and the Reactome database corroborated gene-set enrichments in TuBA’s biclusters including upregulation of cell cycle pathways and downregulation of genes associated with transmission across chemical synapses. Of note, we found the Hedgehog signaling pathway, which has been previously linked to cilium’s role in regulation and signaling (Corbit et al., 2005) to be upregulated in tumors with high cilium expression (Figure 7A). In particular, genes in the hedgehog pathway, including *PTCH2*, *TUBA1C*, *EVC2*, *EVC*, *GLI1*, and *SCUBE2* (DESeq2 FDR <0.001, log fold-change >1) as well as *GLI2*, *GLI3*, and *SMO* (DESeq2 FDR <0.001, log fold-change > 0.5) were upregulated in cilium bicluster samples relative to other tumors (Figure 7B).

**FIGURE 8:**
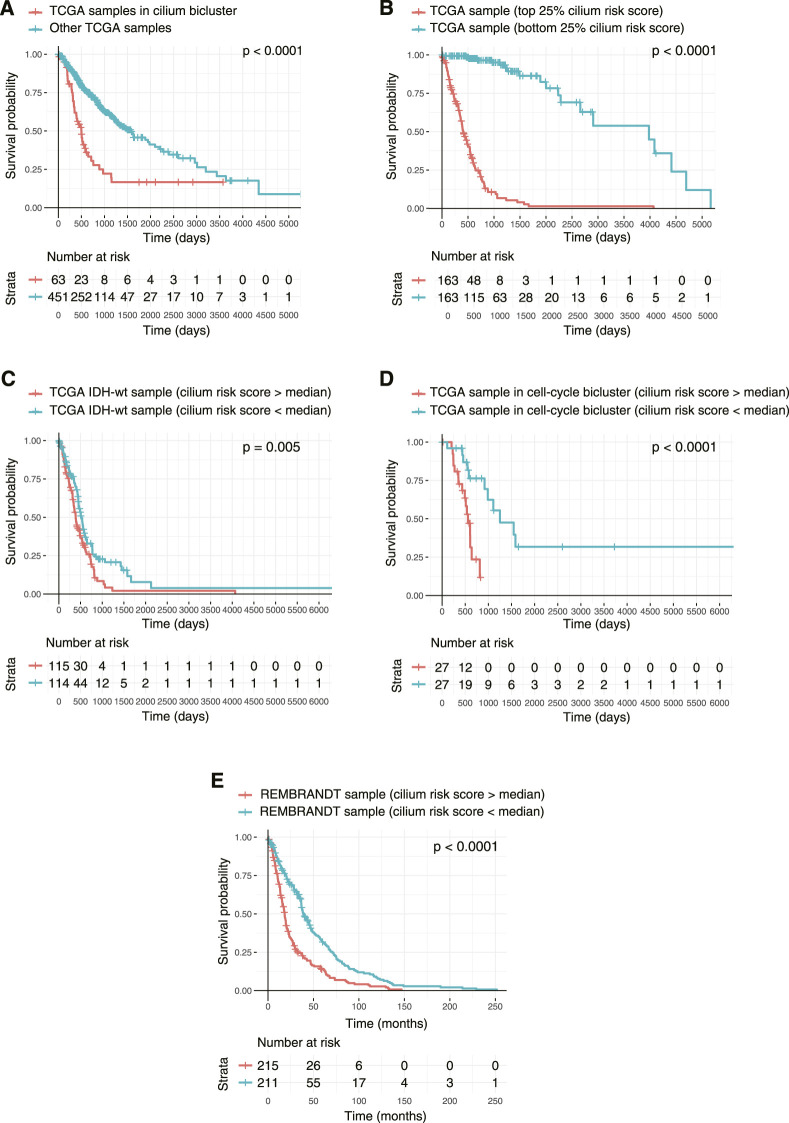
Kaplan-Meier survival analysis of TCGA tumors based on their cilium expression. **(A)** Patients with TCGA tumors in the cilium bicluster had worse overall survival (OS) compared to other patients. **(B)** Patients with tumors in the top 25% cilium risk score had poorer OS compared to patients with tumors in the bottom 25% cilium risk score. **(C)** Patients with *IDH1/2* wild-type gliomas with cilium risk scores below the population’s median had poorer OS compared to those with scores above the median. **(D)** Cilium risk score stratified patient OS in biclusters associated with poor prognosis, including a bicluster enriched in cell cycle. **(E)** Cilium risk score was reproducibly predictive of OS for patients in the independent REMBRANDT cohort.

Finally, to examine the association of the biclusters with prognosis, we conducted Kaplan-Meier analyses and compared the overall survival of patients in each bicluster to all the other patients. The cilium bicluster and 33 others enriched in GBM tumors were associated with poor overall survival (Figure 8A). When we applied TuBA to only the TCGA LGG tumors, among the 93 generated biclusters, once more, we identified a bicluster associated with cilium, which in addition to eight other biclusters was associated with poor overall survival (Supplementary Table S4). Six of these eight biclusters contained genes associated with previously reported prognostic proliferative signature (Phillips et al., 2006).

### A Risk Score Based on Cilium Genes Predicts Patient Survival

Based on our observation that patients with higher expression of cilium-associated genes had worse prognosis, we sought to use these genes for a predictive measure of survival. We built a Cox Proportional-Hazard model based on expression of genes common to the cilium biclusters and included known prognostic factors of patient age at diagnosis, tumor grade, and *IDH1/2* mutated, 1p/19q co-deletion status. Significant features were determined to be grade and presence of *IDH1/2* mutation, 1p/19q co-deletion, and expression of 12 cilium-associated genes: *LRGUK*, *NSUN7*, *LRRC27*, *SPAG17*, *EFHB*, *IFT27*, *DZIP1L*, *FOLR1*, *RGS22*, *TEX9*, *GALNT3*, and *GLB1L*. We then linearly combined these genes to define a cilium risk score, which proved to be indicative of survival, after correcting for other significant factors (Table 1).

**TABLE 1:** Coefficients for calculation of cilium risk score.

Genes	Coefficient
*LRGUK*	0.49
*NSUN7*	0.49
*LRRC27*	−1.50
*SPAG17*	0.25
*EFHB*	−0.31
*IFT27*	−0.87
*DZIP1L*	0.68
*FOLR1*	0.33
*RGS22*	0.31
*TEX9*	−0.15
*GALNT3*	0.46
*GLB1L*	0.84

We asked whether the cilium risk score predicted glioma overall survival. First, we divided the TCGA GBM and LGG samples into two groups: those in the top 25% of the cilium risk score distribution and those in the bottom 25%. The former group had statistically significant decreased overall survival when compared to the latter (Figure 8B). Next, we considered *IDH*-wild-type tumors, which are known to be the clinically poorest performing gliomas. We divided these samples into two groups with high and low cilium risk score based on the median score for this population and observed again that the patients with high-scoring tumors had an overall worse survival when compared to the low-scoring group (Figure 8C). We further showed that median cilium risk score can also stratify patients in the other biclusters associated with poor prognosis, namely those in the cell cycle (proliferative) bicluster (Figure 8D).

Finally, we evaluated the cilium risk score in the independent REMBRANDT glioma dataset, consisting of microarray expression data from 261 GBM to 269 LGG patients (Gusev et al., 2018). Again, patients with higher than the median risk score had a significantly worse overall survival (Figure 8E).

## Discussion

Clustering approaches that group together both genes and samples simultaneously in an unsupervised manner (biclustering) can not only discover genes that are co-expressed aberrantly, but also uncover associations between gene expression and samples’ clinical and genomic attributes. In this study, we present co-regulated gene expression profiles associated with brain normal and tumor tissue. Our unsupervised biclustering analysis using TuBA revealed transcriptional signatures associated with proximally located normal tissue sites and identified distinct co-regulated gene expression profiles associated with tumor grade and patient prognosis. In particular, we identified a set of genes associated with cilium motility that were expressed at a higher level in normal amygdala, hypothalamus, and caudate normal tissue and were aberrantly upregulated in a subset of gliomas. Patients with brain tumors that had high expression of cilium-associated genes had significantly poorer prognosis compared to the rest of the cohort, independent of grade and other clinical and genomic covariates. We then devised a cilium risk score that was reproducibly informative of glioma patient survival after correcting for previously validated, prognostic biomarkers.

Low tumor purity has been previously associated with tumor heterogeneity and highly aggressive phenotypes that arise from the presence of non-tumor and immune cell populations in the microenvironment (Capper et al., 2018; Garofano et al., 2021). It has also been used as a predictor of overall survival for patients with glioma (Zhang et al., 2017). When we compared IHC-based assessments of purity for tumors with and without cilium expression, we did not observe significant differences between the GBM or LGG tumors. However, computational purity inferences by ESTIMATE and LUMP, which utilize gene expression and methylation status respectively, indicated statistically lower purity for the LGG tumors with cilium expression compared to those without. Differential gene expression showed upregulation of 13 and downregulation of three genes included in ESTIMATE scores, suggesting that computational inference of purity may be confounded by aberrant expression of genes that are expected to be only expressed in non-tumor cells.

Cilium expression is not unique to the brain tissue and has been described previously for lung and testis (Patir et al., 2020). It has also been previously explored in the context of tumor development and progression (Han and Alvarez-Buylla, 2010; Liu et al., 2018; Fabbri et al., 2019) particularly for gliomas (Alvarez-Satta and Matheu, 2018; Sarkisian and Semple-Rowland, 2019); however, whether cilium expression aids or hinders tumor proliferation may depend on the tumor type and its genomic and phenotypic attributes (Han et al., 2009; Wong et al., 2009; Zingg et al., 2018). For example, the Smoothened protein, which is localized on cilia and activates the Hedgehog signaling pathway (Bangs and Anderson, 2017), has been shown to trigger transcription of genes related to tumor growth, survival, and the epithelial-to- mesenchymal transition (Liu et al., 2018). Consistently, our differential gene expression and pathway analysis showed upregulation of Hedgehog signaling in tumors with high cilium expression.

The uncovered cilium signature is a reflection of tumor heterogeneity in gliomas. It also suggests a mechanism for their aggressive phenotype, provides a foundation for a reproducible, predictive risk score, and offers potential therapeutic approaches for their targeted treatment (Merchant and Matsui, 2010; Wu et al., 2012; Lu et al., 2021). Although cilium expression is seen in specific regions of normal brain, single-cell-resolution spatial annotation and molecular characterization of gliomas suggest that the origin of cilium signature in tumors may depend on their location and proximity to certain brain regions (Moser et al., 2014; Sarkisian et al., 2014).

## Data Availability

The original contributions presented in the study are included in the article/Supplementary Material, further inquiries can be directed to the corresponding author.
